# Changes in patient-reported outcomes in light chain amyloidosis in the first year after diagnosis and relationship to NT-proBNP change

**DOI:** 10.1038/s41408-021-00412-8

**Published:** 2021-02-01

**Authors:** Anita D’Souza, Ruta Brazauskas, Angela Dispenzieri, Julie Panepinto, Kathryn E. Flynn

**Affiliations:** 1 Division of Hematology/Oncology, Department of Medicine, Medical College of Wisconsin, Milwaukee, WI 53226 USA; 2 Division of Biostatistics, Institute of Health and Safety, Medical College of Wisconsin, Milwaukee, WI 53226 USA; 3 Division of Hematology, Department of Medicine, Mayo Clinic, Rochester, MN USA; 4 Division of Pediatric Hematology/Oncology/BMT, Department of Pediatrics, Medical College of Wisconsin, Milwaukee, WI 53226 USA

**Keywords:** Quality of life, Cancer epidemiology

## Abstract

We conducted a prospective cohort study in newly diagnosed systemic light chain (AL) amyloidosis patients (*N* = 59) to study patient-reported outcomes (PROs) through the first year. The median age was 68 years with 42% female, 8% Black, and 78% lambda subtype. Organ involvement was cardiac in 66%, renal in 58%, with 25% having 3 or greater organs involved. Between baseline and 3 months, all PROMIS^®^-29 domain scores worsened by 0.4–4.1 points except anxiety which improved by 2.1 points. By 1 year, scores improved compared to the greatest decline at 3 months, most statistically significant for global physical health, physical function, and fatigue. On stage-adjusted survival analysis, in addition to baseline global physical and mental health, domains measuring physical function, fatigue, anxiety, depression, and social roles were associated with 1-year survival. At 1 year, PROMIS measures were associated with NT-proBNP changes and hematologic response. Among patients with an NT-proBNP response, the improvement was seen in physical function, social roles, global mental health, and anxiety. Among patients with an NT-proBNP progression, worsening was seen with anxiety, depression, sleep, and global mental health. Measuring and tracking PROs in patients with AL amyloidosis is important and these important outcomes can be used as correlative endpoints in clinical care/research.

## Introduction

Systemic light chain (AL) amyloidosis is a plasma cell neoplasm associated with a multisystem disease with high early mortality and morbidity^1^. Early mortality in the first year of newly diagnosed AL amyloidosis can be as high as 35–40%^2,3^. The strongest known predictor of early mortality is the stage of disease at diagnosis. Stage is currently best determined using the cardiac biomarkers, N-terminal of the prohormone of brain natriuretic peptide (NT proBNP) and troponin T (TnT), and the free light chain burden as determined by the difference between the involved and uninvolved free light chains (dFLC)^4^. Current treatment of AL amyloidosis depends primarily on chemotherapy-based treatment of the amyloidogenic clone to lower the concentration of the involved free light chain. However, this treatment is suboptimal and has no effect on preformed amyloid fibrils for which endogenous clearance is slow. Consequently, it is well recognized that patients may have early symptomatic and biomarker deterioration before improvements, particularly in patients with advanced amyloidosis^5^. Patients with the best organ outcomes are those with rapid clearance of the amyloidogenic free light chain by 90%^6^. Therefore, despite toxicities, chemotherapy treatment is the mainstay of therapy along with supportive care.

Health-related quality of life is an important aspect of AL amyloidosis burden and management. Patient-reported outcome (PRO) measures can be helpful in understanding health-related quality of life and can aid in providing improved care and symptom management^7^. In many diseases, a patient’s health-related quality of life has also been shown to predict clinical outcomes such as overall survival^8,9^.

The primary objective of the current study was to understand the trajectories in PROs in AL amyloidosis in the first year after diagnosis. Additionally, we sought to determine if PRO domains at diagnosis were able to predict early mortality in the first year after diagnosis, after adjusting for other factors predicting survival. Lastly, we assessed the relationships between changes in PRO scores with changes in NT-proBNP, a biomarker that is qualified as a surrogate for survival in AL amyloidosis^10^.

## Materials and methods

We conducted this IRB-approved, prospective cohort study at the Medical College of Wisconsin, Milwaukee (*N* = 35) and Mayo Clinic, Rochester (*N* = 26). Newly diagnosed patients with systemic AL amyloidosis within 3 months of starting treatment were eligible for enrollment. Patients were staged using the Mayo 2004 and 2012 AL amyloidosis staging systems using NT-proBNP, troponin T, and the difference between the involved and uninvolved free light chains^4,11^. We collected PROs from patients in clinic on paper by self-administration at enrollment, 3 months, 6 months and 1-year using the Patient Reported Outcomes Measurement Information System (PROMIS^®^). For patients unable to return to clinic, PROs were completed over the telephone by interviewer administration. At each time point, biomarkers, NT-proBNP, troponin T and dFLC, were also collected in order to assess changes in these measures, and patients were restaged whenever these were available. Date of death or last follow-up was recorded.

### PRO measures

*PROMIS Global Health v1.2* is a 10-item scale covering overall evaluations of physical, mental, and social health, with summary scores for Global Physical Health and Global Mental Health summary scores^12^. In addition, the individual items can be examined separately to provide specific information about perceptions of physical function, pain, fatigue, emotional distress, social health, and general perceptions of health.

*PROMIS-29 Profile v2.0* is comprised of seven 4-item short forms assessing Anxiety, Depression, Fatigue, Pain Interference, Physical Function, Sleep Disturbance, and Ability to Participate in Social Roles and Activities as well as a single Pain Intensity item.

The HealthMeasures Scoring Service was used to score each domain^13^. PROMIS scores are represented on the T-score metric (mean = 50, standard deviation (SD) = 10), and a score of 50 corresponds to the mean of a reference population. The reference population for most PROMIS domains is the general U.S. adult population with the exception of 2 domains, Ability to Participate in Social Roles and Activities and Sleep Disturbance where the calibration sample included more people with chronic illness. Higher scores indicate more of the concept being measured. For example, for the Physical Function domain, a score higher than 50 implies better physical function than average, whereas for Fatigue, a score higher than 50 implies worse fatigue compared to the average of the general US adult population. Minimal important differences vary by domain and the method used to calculate them; with PROMIS measures they may be as low as 2.0–3.0 points (pain, physical function) or 3.0–5.0 points (fatigue)^14,15^. We considered a change of 3–5 points as clinically meaningful a priori based on other work in hematologic malignancies and PROMIS^16^.

### Statistical analysis

Continuous variables were summarized by means and standard deviations while counts and percentages were used for categorical variables. For each PRO domain score, mixed linear models were used to predict changes in mean scores over time (baseline, 3 months, 6 months, and 1 year after enrollment) while accounting for within-subject correlations over time, including all available data from each patient, and considering missing data ignorable conditional on the observed data.

Survival probabilities were estimated using the Kaplan–Meier method. Age, gender, race, stage, AL subtype, number of organs, and type of organ involvement were analyzed as predictors for 1-year survival. Cox proportional hazards regression models were used to assess the relationship between these predictors and survival We then conducted an adjusted survival analysis by introducing each PRO domain as a continuous variable with the significant factors from the survival analysis. A *p*-value of <0.05 was considered statistically significant.

For responsiveness analysis, we used linear regression to calculate the estimate of PRO domain score change corresponding to changes in NT-proBNP. Further, to anchor change in PROs with clinical change, we evaluated the relationship between the percentage change in NT-proBNP and the PRO domain score change. Based on the 2012 criteria for response to treatment^17^, a 30% and >300 ng/L decrease in NT proBNP was considered as a cardiac NT-proBNP response and 30% and >300 ng/L increase as cardiac NT-proBNP progression.

Statistical analysis was conducted using SAS v9 (Cary, NC).

## Results

Of 61 patients enrolled between 2/1/2016 to 4/15/2019, 1 patient was ineligible based on inclusion criteria (the diagnosis of AL amyloidosis was not confirmed) and 1 patient had localized instead of systemic amyloidosis. Among the remaining 59 patients, 44 patients completed the survey prior to starting chemotherapy; of the 15 patients who completed the survey after starting treatment, 11 were within 1 month (including 7 within 7 days and 4 between 7–14 days) and of 4 patients who completed the survey more than a month after starting treatment, 2 were between 1–2 months and 2 between 2–3 months. The baseline characteristics are shown in Table 1. The median follow-up of survivors was 14.4 (range, 2.5–29.8) months. Eighteen patients (31%) underwent an autologous stem cell transplant during the study period including 6 within 3 months. Fifteen patients (25%) died in the first year. An additional 7 patients (12%) were lost to follow-up.

**Table 1:** Baseline characteristics.

	*N* = 59
Age, median (range)	68 (48–83)
Sex (%)	
Male	34 (58)
Female	25 (42)
Race (%)	
White	53 (90)
Black	5 (8)
Declined to answer	1 (2)
AL subtype (%)	
Kappa	13 (22)
Lambda	46 (78)
2004 stage (%)	
I	14 (24)
II	17 (29)
IIIa	18 (30)
IIIb	8 (14)
Missing^a^	2 (3)
2012 stage (%)	
1	9 (14)
2	18 (28)
3	20 (36)
4	10 (16)
Missing^a^	2 (6)
Mean dFLC (SD), mg/L	215.7 (224.6)
Mean NT-proBNP (SD), pg/mL	5310.3 (8445.1)
Mean troponin T (SD), ng/mL(*N* = 34)	0.07 (0.09)
Mean hs troponin T (SD), ng/L (*N* = 23)	12.6 (44)
Cardiac (%)	39 (66)
Renal (%)	34 (58)
Number of organs (%)	
1	21 (36)
2	23 (39)
≥3	15 (25)
First line treatment	
Cylcophosphamide/bortezomib/dexamethasone	44 (75%)
Cyclophsphamide/ixazomib/dexamethasone	2 (3%)
Cylcophosphamide/lenalidomide/dexamethasone	1 (2%)
Bortezomib/dexamethasone	2 (3%)
Daratumumab	1 (2%)
Stem cell transplant	5 (9%)
Unknown	4 (6%)

dFLC—difference between involved and uninvolved free light chains.

^a^Two patients had missing cardiac biomarkers prior to starting therapy.

### Significant change in patient-reported functioning occurs in the first year after diagnosis

**Fig. 1:**
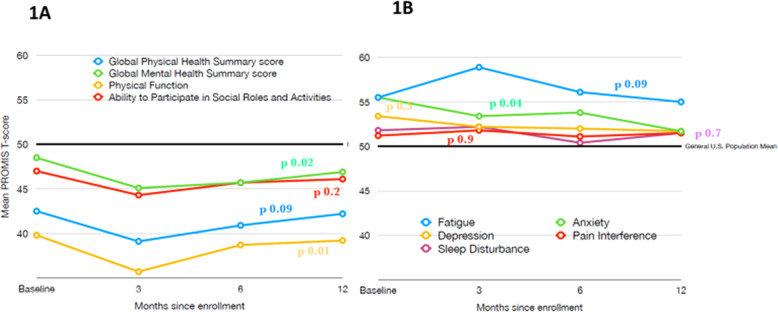
Baseline PRO scores over time. Panel (**A**) shows domain and summary scores where a score below 50 implies worse health/function. Panel (**B**) shows domain scores where a score above 50 implies worse concept being measured.

At 3 months, data were available in 43 patients, at 6 months in 41 patients, and at 1-year in 37 patients (Supplemental Table 1). Table 2 and Fig. 1 show changes in predicted PRO scores over time. Supplemental Table 2 shows the changes in the scores by each time point. Analysis showed significant worsening in multiple PRO domains from baseline to 3 months. A change of ≥3 points was seen in the Global Physical Health Summary (*p*-value 0.02) and Global Mental Health Summary (*p*-value 0.005) scores, as well as the individual domains measuring physical function (*p*-value 0.003) and fatigue (*p*-value 0.03). Additionally, the domain measuring social roles also showed a statistically significant worsening though the change was <3 points (*p*-value 0.04). The only PRO that improved from baseline to 3 months was anxiety (*p*-value 0.04); however, that change was 2.1 points, which did not meet our pre-specified threshold for clinically meaningful change. From 3 months to 6 months, PROs remained relatively stable, though the overall change was toward improvement (Fig. 1). Physical function improved 3.1 points from 3 to 6 months (*p*-value 0.04). At 1 year, PROs were improved compared to the lowest decline at 3 months. This was most evident in magnitude and significance for the Global Physical Health Summary score, which improved from 3 months to 1 year by +3.1 points (p 0.03), Fatigue scores decreased, implying less fatigue, by −3.9 points (*p*-value 0.02) and Physical Function scores improved by +3.5 points (*p*-value 0.02). Table 3 shows the proportion of patients with worse, stable, and improved scores (defined as ≥3 change) for Global Physical Health Summary score, Physical Function, and Fatigue.

**Table 2:** Changes in PROs over the first year.

PROMIS Domain	Baseline mean (SE)	Baseline to 3 months, mean change (*p*-value)	3 months to 1 year, mean change (*p*-value)
Global Physical Health Summary^a^	42.5 (1.6)	−3.4 (***p*** **0.02)**	3.1 (***p*** **0.02**)
Global Mental Health Summary^a^	48.5 (1.2)	−3.4 (***p*** **0.005**)	1.8 (*p* 0.1)
Physical Function^a^	39.8 (1.4)	−4.1 (***p*** **0.002**)	3.5 (***p*** **0.02**)
Ability to Participate in Social Roles and Activities^a^	47.0 (1.4)	−2.8 (***p*** **0.04**)	1.8 (*p* 0.2)
Fatigue^b^	55.5 (1.6)	3.4 (***p*** **0.02**)	−3.9 (***p*** **0.02**)
Anxiety^b^	55.5 (1.1)	−2.1 (***p*** **0.04**)	−1.7 (*p* 0.2)
Depression^b^	53.4 (1.2)	−1.3 (*p* 0.2)	−0.4 (*p* 0.7)
Pain Interference^b^	51.2 (1.4)	0.5 (*p* 0.7)	−0.3 (*p* 0.8)
Sleep Disturbance^b^	51.8 (1.3)	0.4 (*p* 0.8)	−0.7 (*p* 0.6)

Baseline mean T-scores are shown with standard errors and average changes over time with *p*-values (significant *p*-values are in bold).

^a^Negative change implies worsening and positive change improvement for the concept measured.

^b^Negative change implies improvement and positive change worsening for the measured concept.

Bold values indicate statistical significance *p* < 0.05.

**Table 3:** Change in scores in Global Physical Health Summary score, Physical Function, and Fatigue.

Time	Characteristic	Worse	Stable	Improved
At 3 months	Global Physical Health Summary	22 (51%)	16 (37%)	5 (12%)
	Physical Function	23 (53%)	18 (42%)	2 (5%)
	Fatigue	20 (48%)	14 (33%)	8 (19%)
At 6 months	Global Physical Health Summary	21 (53%)	7 (17%)	12 (30%)
	Physical Function	21 (51%)	11 (27%)	9 (22%)
	Fatigue	18 (44%)	13 (32%)	10 (24%)
At 12 months	Global Physical Health Summary	19 (51%)	4 (11%)	14 (38%)
	Physical Function	12 (32%)	19 (51%)	6 (16%)
	Fatigue	15 (41%)	7 (19%)	15 (41%)

Worsening and improvement are defined as ≥3 change in the appropriate direction.

### Survival analysis

One-year survival was 73.5% (95% confidence interval, 59.8–83.2%). Survival analysis (Table 4) showed that stage and number of organs involved significantly predicted 1-year mortality. After adjusting for stage, all domains remained significant except Pain Interference and Sleep Disturbance. Lower Global Physical and Mental Health Summary scores, Physical Function and Ability to Participate in Social Roles and Activities were associated with higher risk of mortality, while increased scores in Anxiety, Depression, and Fatigue were associated with higher risk of 1-year mortality (Table 5).

**Table 4:** One-year univariate survival analysis.

Variable	Categories	Events/N	HR (95% CI)	*p*-value	1-year survival
Sex	Female	5/25	1.00	0.5	80 (58–91)
	Male	10/34	1.49 (0.51–4.35)		69 (50–82)
Race	White	14/53	1.00	0.7	71 (57–83)
	Black	1/5	0.68 (0.09–5.14)		80 (20–97)
AL subtype	Lambda	10/46	1.00	0.1	77 (62–87)
	Kappa	5/13	2.36 (0.80–6.92)		60 (28–81)
2004 stage	I-II	2/31	1.00	0.002	93 (76–98)
	IIIa	7/18	7.24 (1.50–34.88)	0.01	60 (34–79)
	IIIb	6/8	17.46 (3.51–86.88)	0.0005	25 (4–56)
Stage 2012	1–2	1/27	1.00	0.002	96 (76–99)
	3	7/20	10.58 (1.30–86.03)	0.03	62.5 (36–80)
	4	7/10	33.42 (4.09–273.25)	0.001	30 (7–58)
Cardiac AL	No	0/20			100
	Yes	15/39	NE	NE	59 (42–73)
Renal AL	No	6/25	1.00	0.9	76 (54–88)
	Yes	9/34	1.07 (0.38–3.00)		71 (52–84)
Number of organs involved	1	3/21	1.00	0.02	86 (62–95)
	2	4/23	1.24 (0.27–5.57)	0.8	81 (55–92)
	3/+	8/15	5.03 (1.32–19.24)	0.02	39 (12–66)

*NE* non-evaluable.

**Table 5:** Stage-adjusted survival analysis with baseline PROMIS domain score.

PROMIS domain	HR (95% CI)	*p*-value
Global Physical Health Summary^a^	0.5 (0.4–0.8)	0.001
Global Mental Health Summary^a^	0.6 (0.4–0.9)	0.008
Physical Function^a^	0.5 (0.3–0.8)	0.004
Ability to Participate in Social Roles and Activities^a^	0.6 (0.4–0.8)	0.002
Fatigue^b^	1.5 (1.1–2.0)	0.01
Anxiety^b^	1.4 (1.0–2.0)	0.05
Depression^b^	1.7 (1.2–2.5)	0.006
Pain Interference^b^	1.1 (0.9–1.4)	0.4
Sleep Disturbance^b^	1.1 (0.9–1.4)	0.3

The hazard ratio (HR) corresponds to every 5 unit increase in score, adjusted for Mayo Cardiac 2012 stage^4^. HR 1 implies high risk of 1-year mortality.

^a^Domains where higher scores mean improvement in concept being measured.

^b^Domains where higher scores mean worsening in concept being measured.

### Responsiveness of PROMIS related to NT-proBNP changes and hematologic response at 12 months

**Fig. 2:**
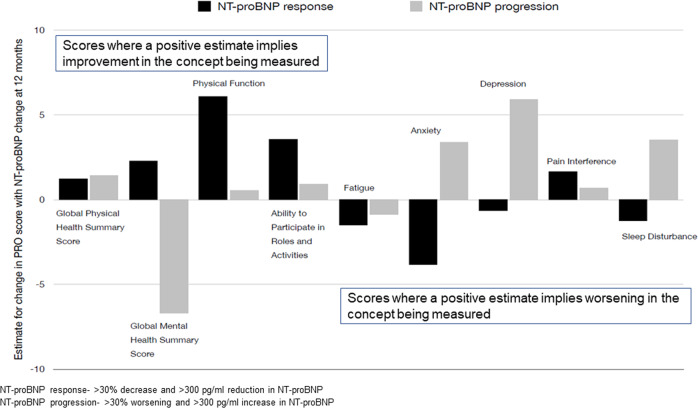
Relationship between NT-proBNP response and change in PRO score at 12 months. The figure shows the relationship between PRO change and NT-proBNP response/progression at 1 year, e.g. for the Physical Function domain, an NT-proBNP response (black bar) results in an improvement in physical function by an estimate of +6.08, whereas an NO-proBNP progression (grey bar) results in change in physical function by an estimate of +0.56. For Anxiety domain, an NT-proBNP response results in an improvement in anxiety (decreased Anxiety scores) by an estimate of −3.84, whereas an NT-proBNP progression results in worsening anxiety (increased Anxiety score) by an estimate of +3.39.

NT-proBNP was available in 33 patients at 6 months and 30 patients at 12 months. For every 1000 pg/ml increase in NT-proBNP, a significantly larger worsening was seen in Global Mental Health Summary, Depression, and Physical Function scores (Table 6). Figure 2 shows the change in PRO domain score by NT-proBNP response and progression. The direction of PRO changes was concordant with NT-proBNP changes and most evident with domains measuring mental function including the Global Mental Health Summary score, Anxiety, Depression, and Sleep Interference. Physical Function and Ability to Participate in Social Roles and Activities showed a large increase with NT-proBNP response while Fatigue showed a modest improvement with NT-proBNP response. The hematologic response was available in 37 patients at 12 months and was shown as very good partial response (VGPR), *N* = 22 or not, *N* = 15. Patients with a VGPR/ > had improved function compared to patients without VGPR across all scores (Supplemental Table 3).

**Table 6:** NT-proBNP response at 12 months and PROs change between baseline and 12 months.

PROMIS domain	Estimate (SE)	*p*-value
Global Physical Health Summary^a^	−0.85 (0.59)	0.2
Global Mental Health Summary^a^	−1.36 (0.45)	0.006
Physical Function^a^	−1.14 (0.40)	0.01
Ability to Participate in Social Roles and Activities^a^	−0.61 (0.46)	0.2
Fatigue^b^	0.64 (0.71)	0.4
Anxiety^b^	1.05 (0.63)	0.1
Depression^b^	1.39 (0.56)	0.02
Pain Interference^b^	0.09 (0.52)	0.9
Sleep Disturbance^b^	1.12 (0.58)	0.06

This table shows the change in PRO domains corresponding to 1000 pg/ml increase in NT-proBNP. The model is adjusted for baseline NT-proBNP (e.g. for 2 patients who start at the same NT-proBNP value, a patient with a 1000 pg/ml increase at 12 months will have 0.85 smaller change in Global Physical Health Summary score than a patient without increase).

^a^Domains where higher scores mean improvement in concept being measured.

^b^Domains where higher scores mean worsening in concept being measured.

## Discussion

Our work examining PROs in systemic AL amyloidosis patients in the first year after diagnosis found that there is significant worsening in PROs in the first 3 months after diagnosis, i.e. during early treatment, improvement in PROs by 1-year; notably in physical function, fatigue, and the Global Physical Summary score, and significant association between baseline PROs with 1-year mortality even after adjusting for amyloid stage. Additionally, PROMIS is responsiveness to clinical change as measured by NT-proBNP response or progression.

The most impaired patient functioning areas at baseline included the global physical health, anxiety, depression, fatigue, physical function, and social roles. We noted a significant improvement in anxiety from diagnosis to 3 months, though the change in score may not be clinically relevant given the <3-point change. Previous work has shown that the anxiety and uncertainty about symptoms that are often associated with a delayed diagnosis may improve after receiving a diagnosis^18,19^. Every other PRO score showed worsening at 3 months. This is consistent with the natural history of AL amyloidosis where a significant proportion of patients experience clinical worsening including the risk of sudden death within the first 3 months after diagnosis^2^. After surviving to the first year after diagnosis, AL patients have a better prognosis with a plateau in survival^4^. We saw similar trends in PRO changes during the first year. The largest change in score as well as most statistically significant changes were seen in PROMIS domains that contributed to the physical health aspect of quality of life- including the Global Physical Health Summary score, Physical Function, Fatigue domains, and less change in Ability to Participate in Social Roles and Activities. Thus, these former domains could be considered the most important to track when using PROs in amyloid clinical research. PROMIS domains measuring mental health, including the Global Mental Health Summary Score, anxiety, depression, and sleep disturbance showed responsiveness to NT-proBNP response at 1-year. Collectively, these results show that, at a minimum, the 10-question PROMIS Global Health scale which provides the Global Physical and Mental Health Summary scores can show measurable changes through the course of the first year to study PRO improvement (or worsening). Further, for a more comprehensive look, the specific domains of interest include those measuring physical function, fatigue, social roles, anxiety, and depression can be measured over time and used to complement other biomarkers of response or progression.

Stage at diagnosis is the most powerful prognostic predictor of 1-year mortality in AL amyloidosis^4^. Thus, when we studied the impact of baseline PROMIS scores on 1 year outcome, we adjusted the model for stage. Our results show that baseline PROs can be helpful in predicting 1-year (early) mortality in AL amyloidosis even after adjusting for stage at diagnosis. The Boston University Amyloid Center has previously shown health-related quality of life to be of prognostic value with worse pre-treatment physical component scores on the SF-36 associated with a greater risk of mortality in patients who received transplant or non-transplant chemotherapy^20,21^. These analyses have shown that the pre-treatment physical component score of the SF-36 can predict post-treatment mortality after adjusting for the number of organs affected^21^. Similarly, the Mayo Clinic has shown that a 3-question screen of patient-reported fatigue, pain, and quality of life can be useful in AL amyloidosis with fatigue independently predicting survival in addition to stage and transplant status^22^. Our results, which build on these previous important studies, elucidate the most important domains that predict 1-year AL survival. In addition to global physical and mental health, PRO domains measuring anxiety, depression, fatigue, physical function, and social roles at diagnosis can predict 1-year mortality after adjusting for stage of disease. We hypothesize that stage, which includes a measure of degree of cardiac involvement, may well adjust for physical domains (e.g. physical function, fatigue) but other domains such as depression and social roles add additional prognostic information that may not be encompassed within the staging system.

In prior analyses of PROs in AL amyloidosis^23,24^, the domain measuring social roles (e.g. Ability to Participate in Social Roles and Activities in PROMIS, Role Physical in SF-36) has been identified as an important domain. At the time of diagnosis, this PROMIS domain was able to discriminate between not only stage of disease, but also cardiac AL involvement, number of organs involved with AL and significantly correlated with NT-proBNP >4,200 pg/ml in a receiver operating characteristic analysis^23^. In the current analysis, we also studied responsiveness of PROMIS to NT-proBNP response/progression at 1-year. While our results are exploratory given small numbers, we found that adverse changes in the domains measuring anxiety, depression, sleep disturbance were associated with NT-proBNP progression while NT-proBNP response was associated with improvements in physical function and anxiety. However, these results should be further explored in larger studies to assess PRO domains being responsive to change in clinical status in AL amyloidosis. The NT-proBNP is considered as an appropriate surrogate endpoint in AL amyloidosis to study change in cardiac AL^10^. However, in a pivotal study of a fibril-directed monoclonal antibody where NT-proBNP was used as the primary endpoint, the study failed to meet this endpoint^25^. Thus, the field of AL amyloidosis continues to seek better biomarkers that can serve as an appropriate endpoint to help in clinical trial design. PROs can be accepted as clinical outcomes assessment tools if they are able to meet the qualifications set by the FDA, and this has been accomplished in other hematologic diseases such as myelofibrosis^26^.

Limitations of our study include a relatively small sample size with loss of patients at each time point. Missing data is an issue in diseases with high mortality with loss of patients at each time point due to death; of the 37% lost at 1 year in our cohort- 25% were due to deaths and only 12% due to attrition not from death. While we used mixed linear models to impute missing PROs, this alone does not account for differences by missing due to death or missing for other reasons. Another issue that was somewhat surprising was that the early mortality of our cohort was ~26%. Early mortality of systemic AL amyloidosis is estimated to be 30–35% based on recent observational data^3^. Many of the patients on the current study from one of the sites were co-enrolled on a prospective clinical trial studying doxycycline which reported an early mortality of 20% at 1-year for these patients^27^. This may have influenced the lower early mortality in our PRO cohort study. In other oncologic settings, the routine tracking of patient-reported symptoms with team follow-up for severe symptoms has led to improved survival^28^, and an alternative hypothesis for the lower mortality in our study is that tracking of PROs led to better symptom management.

These data collectively highlight the importance of measuring and tracking PROs in patients with AL amyloidosis and the potential for using these patient-centered outcomes as important correlative endpoints in designing patient-centered clinical trials to complement existing organ and hematologic biomarkers of change in status. In conclusion, patients with AL amyloidosis have impaired PROs at diagnosis that worsen in the first 3 months and improve for those whose disease improves. In addition, baseline PROs are an independent predictor of one-year mortality for these patients. We conclude that PROs should be utilized consistently in AL amyloidosis to further inform providers and researcher the impact of this disease on patients’ well being.

## Supplementary information

Supplemental material
