# Supplementary material for: Changes in patient-reported outcomes in light chain amyloidosis in the first year after diagnosis and relationship to NT-proBNP change
Source: Blood Cancer J. 2021 Feb 1;11(2):29. doi: 10.1038/s41408-021-00412-8 (PMC7873213; doi:10.1038/s41408-021-00412-8)
Supplement: Supplementary file 1 — Supplemental material [file 41408_2021_412_MOESM1_ESM.docx]

Supplemental table 1. Raw mean T-score with standard deviations.

| **Characteristic** | **Baseline (N=59)** | **3 mo (N=43)** | **6 mo**  **(N=41)** | **1 year (N=37)** | **P-value** |
| --- | --- | --- | --- | --- | --- |
| Global Physical Health Summary* | 42.5 (12.1) | 40.4 (9.2) | 42.2 (9.6) | 43.9 (8.2) | 0.09 |
| Global Mental Health Summary* | 48.5 (9.4) | 45.8 (9.0) | 46.3 (9.6) | 48.0 (9.2) | 0.02 |
| Physical Function* | 39.8 (10.8) | 36.7 (7.7) | 39.6 (8.2) | 41.1 (9.0) | 0.01 |
| Ability to Participate in Social Roles and Activities* | 47.1 (10.9) | 45.2 (8.2) | 46.1 (8.6) | 48.0 (9.3) | 0.2 |
| Fatigue^#^ | 55.6 (12.2) | 57.6 (10.5) | 55.0 (10.0) | 53.6 (8.6) | 0.09 |
| Anxiety^#^ | 55.5 (8.7) | 53.0 (9.4) | 53.3 (10.1) | 51.1 (9.7) | 0.04 |
| Depression ^#^ | 53.4 (9.2) | 50.9 (9.2) | 51.0 (10.2) | 50.7 (7.9) | 0.5 |
| Pain Interference^#^ | 51.2 (10.9) | 52.0 (9.5) | 51.3 (10.2) | 51.4 (10.5) | 0.9 |
| Sleep Disturbance^#^ | 51.8 (9.9) | 52.3 (8.0) | 50.1 (10.6) | 50.5 (9.7) | 0.7 |

*-decreasing score implies worsening and increasing score an improvement for the concept measured

#- decreasing score implies improvement and increasing score a worsening for the measured concept

Supplemental table 2. Comparisons of score change between timepoints. P-values are shown below the score change with standard error.

| **Characteristic** | **0-3 mos** | **3-6 mos** | **6-12mos** | **0-6 mos** | **0-12 mos** | **3-12 mos** |
| --- | --- | --- | --- | --- | --- | --- |
| Global Physical Health Summary* | -3.4 (1.4)  0.0234 | 1.8 (1.6)  0.2554 | 1.3 (1.7)  0.4328 | -1.5 (1.6) 0.3506 | -0.2 (1.3)  0.8618 | 3.1 (1.4)  0.0252 |
| Global Mental Health Summary* | -3.4 (1.2)  0.0052 | 0.6 (1.1)  0.5995 | 1.2 (1.4)  0.3823 | -2.7 (1.1) 0.0167 | -1.5 (1.3)  0.2490 | 1.8 (1.2)  0.1210 |
| Physical Function* | -4.1 (1.3)  0.0025 | 3.1 (1.4)  0.0370 | 0.4 (1.7)  0.7964 | -1.0 (1.6) 0.5172 | -0.6 (1.1)  0.5843 | 3.5 (1.5)  0.0188 |
| Ability to Participate in Social Roles and Activities* | -2.8 (1.3)  0.0385 | 1.4 (1.4) 0.3117 | 0.4 (1.5)  0.7919 | -1.4 (1.6) 0.3982 | -1.0 (1.2)  0.4333 | 1.8 (1.3)  0.1844 |
| Fatigue^#^ | 3.4 (1.5)  0.0251 | -2.9 (1.8)  0.1246 | -1.0 (1.7)  0.5519 | 0.5 (1.7) 0.7580 | -0.5 (1.5)  0.7369 | -3.9 (1.7)  0.0241 |
| Anxiety^#^ | -2.1 (1.0)  0.0360 | 0.4 (1.3)  0.7762 | -2.1 (1.3)  0.1145 | -1.7 (1.2) 0.1437 | -3.8 (1.4)  0.0074 | -1.7 (1.2)  0.1733 |
| Depression ^#^ | -1.3 (0.9)  0.1886 | -0.1 (1.2)  0.9181 | -0.3 (1.5)  0.8327 | -1.4 (1.3) 0.2884 | -1.7 (1.2)  0.1749 | -0.4 (1.1)  0.6873 |
| Pain Interference^#^ | 0.5 (1.2)  0.6637 | -0.7 (1.1)  0.5405 | 0.4 (1.2)  0.7339 | -0.2 (1.3) 0.9056 | 0.2 (1.4)  0.8636 | -0.3 (1.0)  0.7894 |
| Sleep Disturbance^#^ | 0.4 (1.5)  0.7663 | -1.9 (1.6)  0.2344 | 1.2 (1.6)  0.4741 | -1.4 (1.5) 0.3451 | -0.3 (1.3)  0.8204 | -0.7 (1.4)  0.6116 |

*- negative change implies worsening and positive change improvement for the concept measured

#- negative change implies improvement and positive change worsening for the measured concept

Supplemental Table 3. Comparison of 12-month PRO score by 12-month hematologic response status.

| **PROMIS domain** | **No VGPR (n=22)** | **VGPR (n=15)** |
| --- | --- | --- |
| Global Physical Health Summary* | 41.9 (6.6) | 46.9 (9.6) |
| Global Mental Health Summary* | 45.6 (6.8) | 51.6 (11.1) |
| Physical Function* | 40.1 (8.3) | 42.5 (9.9) |
| Ability to Participate in Social Roles and Activities* | 46.7 (9.1) | 50.0 (9.6) |
| Fatigue^#^ | 54.4 (7.8) | 52.4 (9.8) |
| Anxiety^#^ | 52.1 (9.7) | 49.6 (9.7) |
| Depression^#^ | 52.2 (7.5) | 48.6 (8.1) |
| Pain Interference^#^ | 54.2 (10.7) | 47.6 (9.2) |
| Sleep Disturbance^#^ | 53.3 (9.8) | 46.3 (8.3) |

*-lower score implies worse and higher score improvement for the concept measured

#- lower score implies improvement and higher score worsening for the measured concept
